# Laguerre-Gaussian modes generated vector beam via nonlinear magneto-optical rotation

**DOI:** 10.1038/s41598-021-85249-8

**Published:** 2021-03-16

**Authors:** Mohsen Ghaderi Goran Abad, Mohammad Mahmoudi

**Affiliations:** Department of Physics, University of Zanjan, University Blvd., Zanjan, 45371-38791 Iran

**Keywords:** Magneto-optics, Nonlinear optics

## Abstract

Laguerre-Gaussian (LG) beams contain a helical phase front with a doughnut-like intensity profile. We use the LG beam to introduce a rather simple method for generation of a vector beam (VB), a beam with spatially-dependent polarization in the beam cross section, via the nonlinear magneto-optical rotation (NMOR). We consider the NMOR of the polarization of a linearly polarized probe field passing through an inverted Y-type four-level quantum system interacting with a LG control field and a static magnetic field. It is shown that the polarization of the transmitted field is spatially distributed by the orbital angular momentum (OAM) of the LG control field, leading to generation of the VB with azimuthally symmetric polarization distribution. We show that the polarization and intensity distributions of the VB spatially vary by changing the OAMs of the LG control field. Moreover, the radial index of the LG control field has a major role in more spatially polarization distributing of the VB. It is shown that the intensity of the generated VBs in different points of the beam cross section can be controlled by the OAM as well as the radial index of the LG control field. However, the VB with highly spatially distributed can be generated for higher values of the radial index of LG control field. The analytical calculations determine the contribution of the different nonlinear (cross-Kerr effect) phenomena on the generation of the VB. We show that the VB is mainly generated via birefringence induced by the applied fields. Finally, we use asymmetric LG (aLG) beams for making the VBs with asymmetric polarization distribution. It is shown that by applying aLG beams, the azimuthal symmetry of the polarization distribution breaks and the asymmetric polarization distribution can be controlled by OAM and radial index of the aLG control field. The obtained results may find more interesting applications in fiber/free space optical communication to enhance the capacity of the information transmission.

## Introduction

Polarization is a fundamental property of light and a significant concept in optics. Specification and manipulation of polarization of light plays an important role in light-matter interaction^1–4^. Three well-known polarization states of a polarized light such as linear, circular, and elliptical are uniformly spatially distributed. Most past research in polarization dealt with spatially homogeneous states of polarization, which do not depend on the spatial location in the beam cross section. Recently, because of interesting properties and potential applications, there has been an increasing attention to light beams with spatially-dependent polarization in beam cross section, the so-called vector beams. Vector beams (VBs) have spatially variant polarization states with the annular intensity distributions^5^. It has been demonstrated that VBs have significant features including tight focusing^6–8^ and high-resolution imaging^9–11^. The ability of tight focusing of VBs and generating strong longitudinal electric field components within the focus lead VBs to be used in optical trapping and manipulation^12–14^. In addition, VBs hold a large potential for data storage and quantum information processing^15,16^. For decades, amplitude, frequency, phase and polarization of light were the traditional degrees of freedom of light in optical communications, leading to impose some limits on the capacity of the information transmission. By introducing the beams carrying the orbital angular momentum (OAM) with helical phase front, an additional degree of freedom was provided for photons and hence a set of higher dimensions is presented for the high capacity information transmission^17,18^. VBs have attracted significant attention in increasing the transmission capacity in optical communications due to exploiting spatial polarization structure^19^. Unique properties and extensive applications of VBs have motivated researchers to propose various methods to generate VBs. The common methods include using optical fibers^20,21^, spatial light modulator^22,23^, arrays of concentric nanoslits^24,25^ and Pancharatnam-Berry phase elements^26^. Circularly polarized fields have been often used to generate VB through metallic structures including nanometers unit^27^ and metasurfaces^28,29^. To the best of our knowledge, linearly polarized light has been rarely used due to the complexity of its conversion to VBs. The known mechanism of the NMOR in our work provides a simple understanding for converting a linearly polarized light to a controllable VB. Moreover, the volume of optical devices in the previously presented methods is large with a complex experimental setup, while the NMOR has generally a rather simple setup with stable output.

On the other hand, polarization rotation of a polarized light has been receiving much attention for a wide variety of its applications for many decades. It is well-known that when a linearly polarized light^30^ or even elliptically polarized light^31^ pass through an anisotropic medium, the light polarization plane experiences a rotation. NMOR arises when the polarization plane of light is rotated by a medium subjected to a magnetic field and laser fields. In fact, the difference between the refractive indices of the circular components of the linearly polarized light is the basis of the asymmetry made by the magnetic or optical fields. NMOR has found a large number of applications^32^ and been used as a practical and useful method in optical filters^33,34^, optical limiting^35,36^, magnetometry^37–39^ and laser-frequency stabilization^40^.

In the past three decades, of among all vortex beams, Laguerre-Gaussian (LG) beams are the most interesting due to their unique features in a wide variety of applications^41–44^. LG modes are obtained from solving the paraxial Helmholtz equation in cylindrical coordinates. The azimuthal phased dependence ($e^{il\phi }$) of the LG modes leads to carrying OAM by $l\hslash$ per photon^45^, where *l* is an integer. LG modes have a helical wave front with a quantized $2\pi l$ azimuthal phase change of the electric field. Moreover, the phase singularity of the LG modes on the beam axis dictates zero intensity at the beam center^46^, leading the intensity pattern of the LG modes to take the form of a doughnut or even concentric rings. It has been demonstrated that the characteristics of the optical phenomena can be influenced by the LG beams^47–49^. Mahmoudi et al. showed that the use of LG beams narrowed the linewidth of the optical spectrum of the multi-photon resonance phenomena^50^. However, what caught our eye was the observation of the spatial dependence of some optical phenomena like electromagnetically induced transparency^51^ and entanglement^52^ using vortex light beams. It inspired us to impart the potential of LG modes to the NMOR for obtaining the distribution of polarization, leading to generate VBs. In this regard, we implement the nonlinear magneto-optical rotation (NMOR) using the LG beams as a rather simply novel technique to the generation of VBs.

In this paper, we propose the NMOR as a new simple technique to generate VBs and control their spatial polarization distribution. The aim of our work is to simplify the generation and control of VBs with respect to the previous works. The presented scheme includes the NMOR of a linearly polarized probe field passing through an inverted Y-type four-level quantum system subjected to a LG control field and a static magnetic field. It is demonstrated that the OAM of the LG control field makes the polarization of the transmitted field to be spatially distributed, leading to the generation of VBs with azimuthally symmetric polarization distribution. We show that by increasing the magnitude of the OAM, the spatial distribution of VBs varies and their polarizations are more distributed in cross section of the VBs. It is illustrated that the radial index of the LG control beam has a major role in changing the polarization directions and the spatially distribution of VBs. In addition, we demonstrate that the intensity of the generated VBs can be simply controlled by the characteristics of the LG beam. However, VBs with more higher intensity regions are generated by increasing the magnitude of the radial index of the LG beam. Our analytical results show the role of the direct response and multi-photon nonlinear cross-Kerr effect in generation of the VBs. We demonstrate that the polarization rotation in different points of the VB cross section is related to the major contribution of the birefringence induced in the system. Finally, we exploit asymmetry LG (aLG) beams and breaks the symmetry of the polarization distribution of the VBs. It is shown that by applying the aLG control field, azimuthally asymmetric polarization distribution is achieved so that the asymetric polarization distribution can be controlled by OAM and radial index of the aLG control field. Generating and controlling the spatial distribution of VBs in our work provide an excessive capacity in optical communicating and networking.

## Model and theoretical method

**Figure 1:**
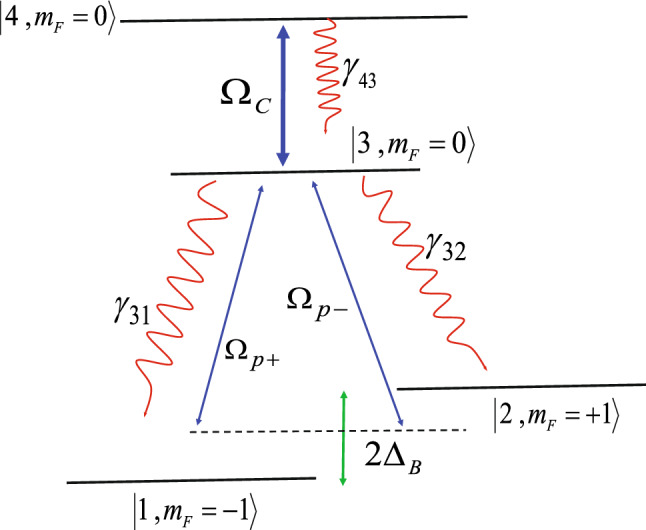
Schematic diagram of an inverted Y-type four-level quantum system. The system is driven by a LG control field with Rabi frequency $\Omega _{c}$, a left- and right-circularly polarized fields with Rabi frequencies $\Omega _{p-}$ and $\Omega _{p+}$ , respectively, derived from a linearly polarized probe field.

The realistic quantum system of interest is shown in Fig. 1. We consider an inverted Y-type four-level quantum system, which can be derived from $5S_{1/2}-5P_{3/2}-5D_{5/2}$ lines of $^{87}Rb$ atoms in a vapor medium. Two states $|1\rangle =|5S_{1/2},(F=2,m_{F}=-1)\rangle$ and $|2\rangle =|5S_{1/2},(F=2,m_{F}=+1)\rangle$ are the degenerate ground states. The state $|3\rangle =|5P_{3/2},(F=3,m_{F}=0)\rangle$ is set as intermediate state and the state $|4\rangle =|5D_{5/2},(F'=2,m_{F'}=0)\rangle$ is assumed as the excited state. Here, *F* and $F'$ are the quantum numbers of the total angular momentum and also $m_{F}$ denotes the magnetic quantum number of the corresponding states. In the considered system, the Doppler effect due to the motion of atoms is ignored. A static magnetic field is applied to the system, which lifts the degeneracy of the ground states by $\hslash \Delta B=m_{s}g_{s}\mu _{B}B$ (Zeeman splitting) where $\mu _{B}$ is Bohr magneton, $g_{s}$ is Lande’ factor and $m_{s}=\pm 1$ is the magnetic quantum number of the corresponding sublevel of the ground state. A linearly polarized weak probe field $\vec {E}={\hat{x}} E_{p} exp[-i(\omega _{p}t-k_{p}z)]+c.c$ with a wavelength of 780.238 nm is applied to the medium parallel to the static magnetic field satisfying the Faraday geometry^53^. A linearly polarized field is composed of a right- and left-circularly polarized component. Then, right-(left-) circular component of the probe field excites the transition $|3\rangle \leftrightarrow |1\rangle$($|3\rangle \leftrightarrow |2\rangle$) with Rabi frequency $\Omega _{p+}=(\vec {\mu }_{31}\cdot \vec {\epsilon }_{+})E_{+}/\hslash$ ($\Omega _{p-}=(\vec {\mu }_{32} \cdot \vec {\epsilon }_{-})E_{-}/\hslash$), so that $E_{+}=E_{-}=E_{p}/\sqrt{2}$ and $|\vec {\mu _{41}}|=|\vec {\mu _{31}}|$. Also, a linear polarized LG control field with a wavelength of 775.978 nm couples the intermediate state $|3\rangle$ to the excited state $|4\rangle$ with Rabi frequency $\Omega _{c}=(\vec {\mu }_{43} \cdot \vec {\epsilon }_{c})E_{c}/\hslash$. Here, $\epsilon _{c}$ is the coupling field unit vector and $\epsilon _{\pm }$ are the right- and left-rotating unit vector and $E_{i}(i=\pm ,c)$ are the amplitudes of the applied fields. Moreover, $\mu _{ij}$ is the dipole moment vector for the transitions $|i\rangle$ to $|j\rangle$. The LG control field in the cylindrical coordinates has the form

1 $$\begin{aligned} E_{c}(r,\varphi )= E_{0_{c}}\frac{w_{G}}{\sqrt{|l|!}w_{LG}} \left(\frac{\sqrt{2}r}{w_{LG}}\right)^{|l|}\times L^{|l|}_{p}(x)e^{-r^{2}/w_{LG}^{2}}e^{il\varphi }, \end{aligned}$$

where $E_{0_c}$, *l* and *p* denote the amplitude, OAM and radial index of the LG control field, respectively. $w_{G}$ and $w_{LG}$ are the Gaussian and LG beam waist, respectively. $L^{|l|}_{p}(x)$ with $x=2r^{2}/w_{LG}^{2}$ is the Laguerre polynomial, which is written as

2 $$\begin{aligned} L^{|l|}_{p}(x)=\frac{e^{x}x^{-|l|}}{p!}\frac{d^p}{dx^p} \left[x^{|l|+p}e^{-x}\right]. \end{aligned}$$

In the interaction picture, the Hamiltonian of the system in the dipole and rotating wave approximations is written as

3 $$\begin{aligned} V_{I}=-\hslash (\Omega ^{*}_{p+}e^{i(\Delta _{p+}+\Delta _{B})t}|3\rangle \langle 1|+\Omega ^{*}_{p-}e^{i(\Delta _{p-}-\Delta _{B})t}|3\rangle \langle 2|+\Omega ^{*}_{c}e^{i\Delta _{c}t}|4\rangle \langle 3|)+h \cdot c., \end{aligned}$$

where $\Delta _{p+}=\omega _{31}-\omega _{p+}$, $\Delta _{p-}=\omega _{32}-\omega _{p-}$ and $\Delta _{c}=\omega _{43}-\omega _{c}$ are the detunings of the applied fields from the corresponding atomic transitions. The terms $\omega _{p+}$, $\omega _{p-}$ and $\omega _{c}$ are the frequencies of the right-, left-circular components and control field, respectively. Also, $\omega _{ij}$ is the $|i\rangle \leftrightarrow |j\rangle$ atomic transition frequency.

The density matrix equations of motion are given by

4 $$\begin{aligned} {\dot{\rho }}_{11}&= \gamma _{31}\rho _{33}-i\Omega ^{*}_{p+}\rho _{13}+i\Omega _{p+}\rho _{31},\nonumber \\ {\dot{\rho }}_{22}&= \gamma _{32}\rho _{33}-i\Omega ^{*}_{p-}\rho _{23}+i\Omega _{p-}\rho _{32},\nonumber \\ {\dot{\rho }}_{44}&= -\gamma _{43}\rho _{44}-i\Omega _{c}\rho _{43}+i\Omega ^{*}_{c}\rho _{34},\nonumber \\ {\dot{\rho }}_{31}&= -[\gamma _{3}/2+i(\Delta _{p+}+\Delta _ B)]\rho _{31}+i\Omega _{p-}\rho _{21}+i\Omega _{c}\rho _{41}+i\Omega ^{*}_{p+}(\rho _{11}-\rho _{33}),\nonumber \\ {\dot{\rho }}_{41}&= -[\gamma _{43}/2+i(\Delta _{c}+\Delta _{p+}+\Delta _ B)]\rho _{41}+i\Omega ^{*}_{c}\rho _{31}-i\Omega ^{*}_{p+}\rho _{43},\nonumber \\ {\dot{\rho }}_{32}&= -[\gamma _{3}/2+i(\Delta _{p-}-\Delta _ B)]\rho _{32}+i\Omega ^{*}_{p+}\rho _{12}+i\Omega _{c}\rho _{42}+i\Omega ^{*}_{p-}(\rho _{22}-\rho _{33}),\nonumber \\ {\dot{\rho }}_{42}&= -[\gamma _{43}/2+i(\Delta _{c}+\Delta _{p-}-\Delta _ B)]\rho _{42}+i\Omega ^{*}_{c}\rho _{32}-i\Omega ^{*}_{p-}\rho _{43},\nonumber \\ {\dot{\rho }}_{43}&= -[(\gamma _{3}+\gamma _{43})/2+i\Delta _{c}]\rho _{43}-i\Omega _{p-}\rho _{42}-i\Omega _{p+}\rho _{41}+i\Omega ^{*}_{c}(\rho _{33}-\rho _{44}),\nonumber \\ {\dot{\rho }}_{21}&= -i(\Delta _{p+}-\Delta _{p-}+2\Delta _{B})\rho _{21}-i\Omega _{p+}\rho _{23}+i\Omega _{p-}\rho _{31},\nonumber \\ {\dot{\rho }}_{33}&= -({\dot{\rho }}_{11}+{\dot{\rho }}_{22}+ {\dot{\rho }}_{44}), \end{aligned}$$

where $\gamma _3=\gamma _{31}+\gamma _{32}$ . Parameter $\gamma _{43}$ is the decay rate from the excited state $|4\rangle$ to the intermediate state $|3\rangle$ and $\gamma _{3i}=\gamma (i=1,2)$ is the decay rate of the intermediate state $|3\rangle$ to the ground state $|i\rangle$. The susceptibility of the medium corresponding to the right- and left-circular components of the linearly polarized probe field is given by

5 $$\begin{aligned} \chi _{\pm }= \left (\frac{\alpha }{4\pi k_{p}}\right)S_{\pm }, \end{aligned}$$

where $k_p$ is the wave number of the probe field and $\alpha l=4 \pi k_pd\mu ^2 N/\hslash \gamma$ is the field absorption at resonance,where *N* and *d* are the atomic density number and length of the medium, respectively. We introduce $S_{\pm }$ as normalized susceptibility

6 $$\begin{aligned} S_{+}=\frac{\rho _{31}\gamma _{31}}{\Omega _{p+}}, \quad \quad \quad S_{-}=\frac{\rho _{32}\gamma _{32}}{\Omega _{p-}}. \end{aligned}$$

$\rho _{31}$ and $\rho _{32}$ are the transition coherences, which can be obtained from Eq. (4). The term $S_{\pm }$ is a complex quantity, which its real (imaginary) part indicates the dispersion (absorption) of the circular components of the probe field. Now, let us calculate the NMOR angle of the polarization direction of the transmitted VBs. It should be noted that the polarization direction of the input probe field is assumed in *x* direction. In experimental works, the polarization rotation can be measured using a ${\hat{y}}$-polarized analyzer permitting only the polarized field in ${\hat{y}}$ direction. Intensity of transmission of VB with polarization direction in *y* ($T_y$) and *x* ($T_x$) is given by^30^

7 $$\begin{aligned} T_{y}= & {} \frac{|(E_{p_{(out)}})_{y}|^2}{|E_{p_{(in)}}|^2}=\frac{1}{4}|exp[i\alpha l S_{+}/2]-exp[i\alpha l S_{-}/2]|^2 \end{aligned}$$

8 $$\begin{aligned} T_{x}= & {} \frac{|(E_{p_{(out)}})_{x}|^2}{|E_{p_{(in)}}|^2}=\frac{1}{4}|exp[i\alpha l S_{+}/2]+exp[i\alpha l S_{-}/2]|^2 \end{aligned}$$

Thus, the NMOR angle of the polarization direction of the VBs is

9 $$\begin{aligned} \phi =tan^{-1}[\sqrt{T_{y}/T_{x}}]. \end{aligned}$$

In general, rotation of the polarization direction of a polarized light happens due to birefringence and dichroism induced in the system. Difference between dispersion (absorption) of the circular components of the probe field leads to inducing the birefringence (dichroism) in the system. For the case that $Re[S_{+}]\ne Re[S_{-}]$ and $Im[S_{+}]= Im[S_{-}]\approx 0$, the Polarization direction of light is rotated merely due to the birefringence. On the contrary, dichroism is the dominant phenomenon when $Im[S_{+}]\ne Im[S_{-}]$ and $Re[S_{+}]= Re[S_{-}]\approx 0$.

## Results and discussion

**Figure 2:**
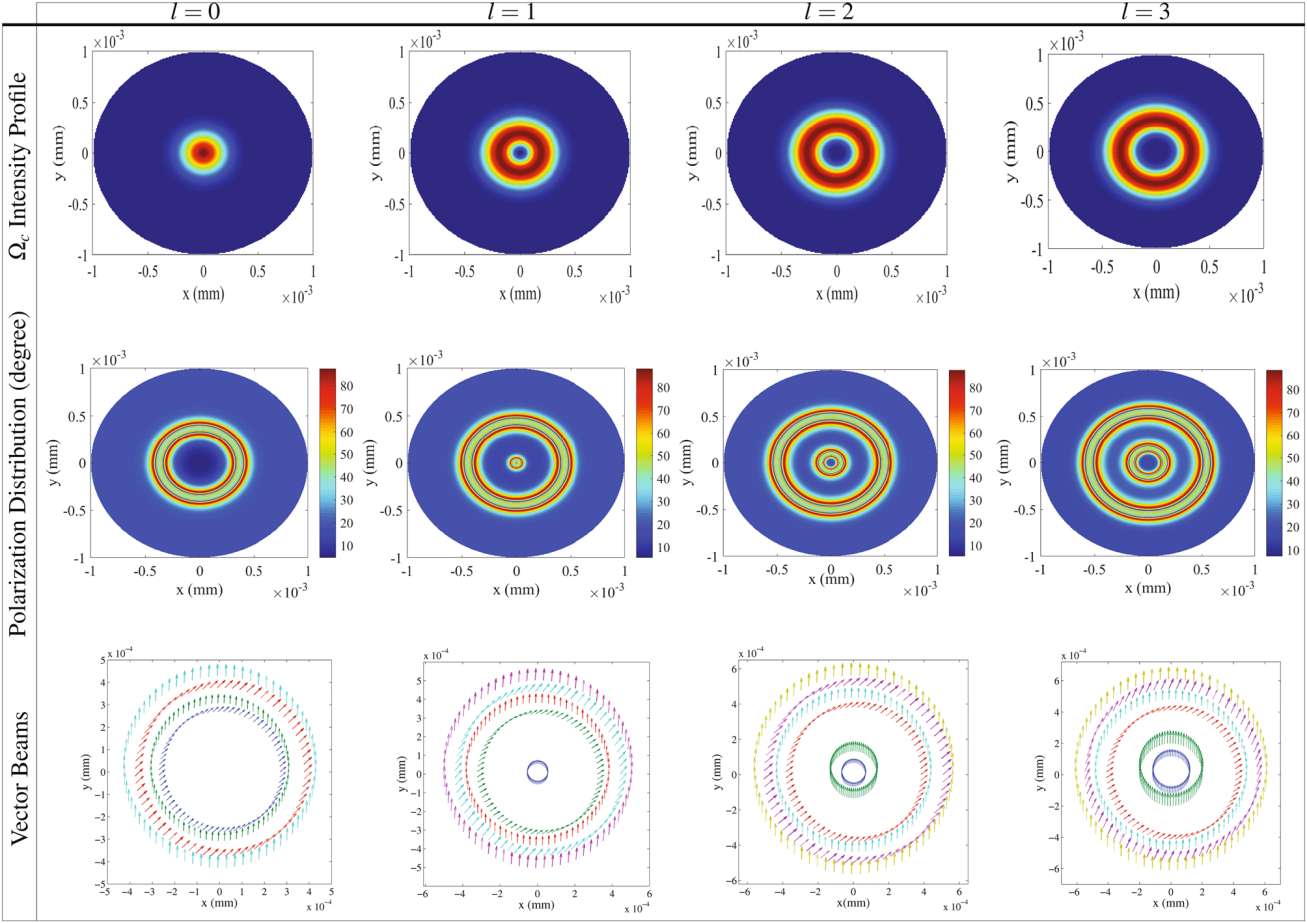
The intensity profile of the Gaussian and LG control field (first row), polarization distribution of the transmitted probe field (second row) in unit of degree and a schematic of the generated VBs (third row) as a function of *x* and *y* for Gaussian field $l=0$ and different modes of LG control field $l= 1$, $l= 2$ and $l= 3$. Used parameters are $\Omega _{0c}= 18\gamma$, $\Omega _{p}= 0.01 \gamma$, $\Delta _{B}= 10 \gamma$, $\alpha l= 140 \gamma$, $\Delta _c =0$, $\Delta _p= 0$, $w_G= 1 \; \text{mm}$, $w_{LG}= 270 \; \text{mm}$ and $p=0$.

Now, we are going to study the distribution of the polarization of the transmitted probe field and generation of VBs through the NMOR by numerically solving the Eq. (4) in steady state condition. It is well known that the transmission of the probe field passing through the medium can be affected by intensity of the coupling field via the nonlinear cross-Kerr effect. We are going to use this fact to generate the light beams with spatially-dependent polarization in the beam cross section. Throughout the results, it is assumed that $\Delta _-=\Delta _+=\Delta _p$ and $\Omega _{p-}=\Omega _{p+}=\Omega _p$. Also, the parameters used are scaled with $\gamma$ which is taken as $\gamma = 2\pi \times 6$ MHz for $D_2$ transition of $^{87}$Rb. Here, we are interested in investigating the NMOR angle of the linearly polarized probe field in the cross section of the transmitted probe field which is called the spatial polarization distribution. Figure 2 shows the intensity profile of the Gaussian and different modes of the LG control field (first row), the spatial polarization distribution of the transmitted field (second row) in unit of degree and a schematic of the corresponding VBs (third row) as a function of *x* and *y* for the Gaussian and different modes of the LG control field. Used parameters are $\Omega _{0c}=(\vec {\mu }_{43} \cdot \vec {\epsilon }_{c})E_{0c}/\hslash = 18\gamma$, $\Omega _{p}= 0.01 \gamma$, $\Delta _{B}= 10 \gamma$, $\alpha l= 140 \gamma$, $\Delta _c =0$, $\Delta _p= 0$, $w_G= 1\; \text{mm}$, $w_{LG}= 270 \; \text{mm}$ and $p=0$. The first row of Fig. 2 shows the intensity distribution of the Gaussian and LG control field cross section affected by the OAM. Because of the spatially-dependent intensity profile of the LG control field, it is expected that the LG control field induces the spatially-dependent transmission for the right- and left-circular components of the probe field. The second row describes that the polarization of the transmitted probe field is spatially distributed and the spatially homogeneous state of polarization of the probe field switches to the VB. It is seen that the polarization direction of the generated VB experiences the NMOR form zero to 90° at different points of a cross section of the probe beam. For the Gaussian control field, there are only two rings with perfect NMOR angle accompanied by a large area with negligible rotation angle at the center of beam shown in first column of Fig.2. Moreover, the rest area in the cross section possesses spatially different distribution of the NMOR angle. Now, we apply the LG control field containing OAM with different topological charges, i.e $l=1$ (second column), $l=2$ (third column) and $l=3$ (fourth column) in Fig. 2. Applying different modes of the LG control field generates the various spatial distribution of the polarization. It is observed that four rings appear with perfect NMOR angle and the rest area has differently spatial distribution of the polarization. The new two rings appeared nearer the center of the profiles do not exist in the case of the Gaussian control field. Note that the inner ring for $l=1$ in the second column is not shown because of its smaller radius with respect to the other three rings. It is shown that by increasing the OAM of the LG control field, the polarization distribution spatially varies in the cross section of the probe field and is extended to larger radii. These results are in good agreement with increasing the radius of maximum optical intensity for higher LG modes^54^. A schematic of the corresponding VBs is presented in the third row of Fig. 2. Every vector stands for a local electric field direction at a cross section of the probe field. Since the polarization direction of the input field has been assumed in *x* direction, the angle between any vector beam and axis *x* indicates the amount of the NMOR of the polarization direction of the linearly polarized probe field. Figure 2 clearly shows that although the input probe field has a homogeneous polarization in *x* direction, the output probe field after passing through the medium has a various polarization in different points of the cross section of the probe field, leading to generation of the VB. Thus, the generation, spatial distribution and polarization directions of the VBs depend on the topological charge of the LG control field.

**Figure 3:**
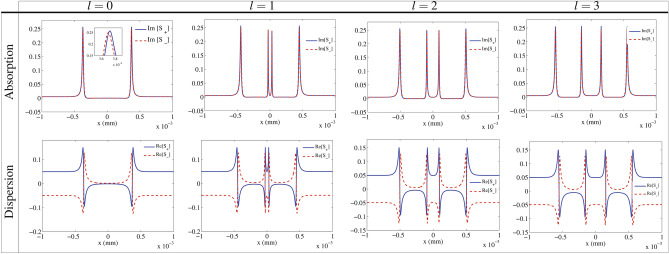
The Absorption (first row) and dispersion (second tow) of the right-(solid) and left-(dashed) circular components of the linearly polarized probe field in one dimension (*x*). The used parameters are those taken in Fig. 2.

To clarify the mechanism of the MORs happened in Fig. 2, we are interested in investigating the absorption and dispersion spectrum of the circular components of the input field. The absorption (first row) and dispersion (second row) of the right- (solid) and left-(dashed) circular components of the transmitted probe field are depicted in one-dimension *x* in Fig. 3. It is shown in the first row that the absorptions of the circular components of the linearly polarized probe field are approximately the same in most regions of the VB’s cross section. There are only two peaks in absorption spectrum for the Gaussian beam and four peaks for different modes of the LG control fields, while the absorption is negligible in the rest regions for both circular components. Although there is a significant absorption in the peaks for circular components, the amount of their difference is still negligible. The second row of Fig. 3 shows that the dispersion is largely different in most areas of the VB’s cross section for the right- and left-circular components. Difference dispersion of the circular components accompanied by their negligible absorption difference leads to inducing the birefringence in all parts of the cross section. Thus, the MOR happened at different points of the cross section is mainly due to birefringence induced in the system.

**Figure 4:**
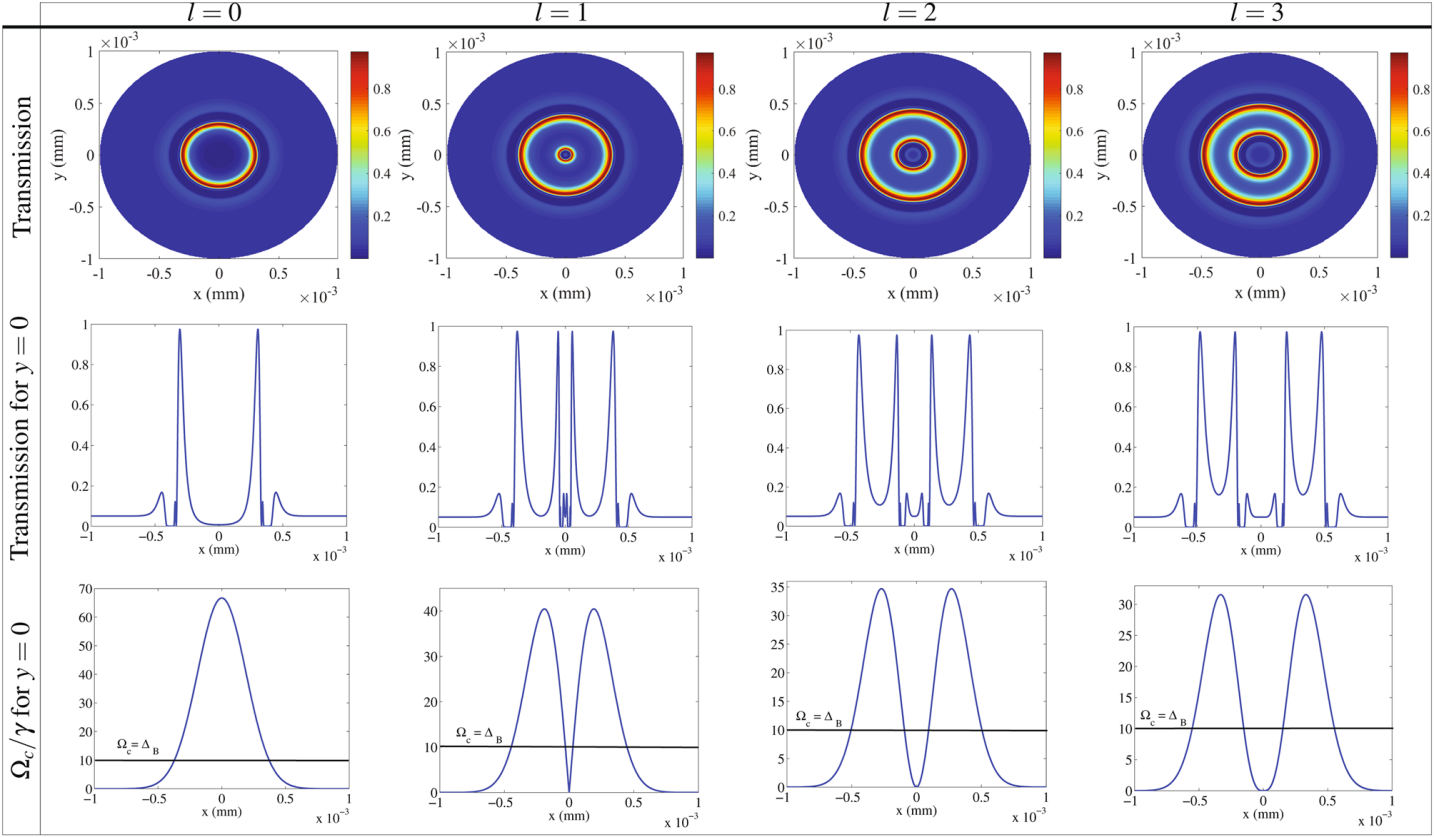
The *y*-polarized intensity distribution of VBs related to the Fig. 2 as a function of *x* and *y* (first row), the perspective of transmission in $y=0$ (second row) and the evolution of the Rabi frequency of the LG control field for $y=0$ (third row). The used parameters are those used in Fig. 2.

Let us study the intensity of the output VB, which has a major role in VB transmission. In this regards, the *y*-polarized transmission distribution of the generated VB is depicted in the first row of Fig. 4 as a function of *x* and *y* for different values of the OAM, i.e. i.e $l=0$ (first column), $l=1$ (second column), $l=2$ (third column) and $l=3$ (fourth column). The taken parameters are those used in Fig. 2. It is shown that the generated VBs are transmitted with different intensity profiles, depending on the OAM of the LG control field. An investigation on the first row of the Fig. 4 displays various transmissions for different radii of the VBs cross section, so that it can be found the transparent rings with locations depending on the OAM of the LG control field. Figure 4 shows that the transmissions behavior in four higher NMOR rings, displayed in Fig. 2, are physically different and only two rings with higher intensity of transmissions appear in the transmission profile. The second row in Fig. 4 shows the perspective of transmission in $y=0$. One can see the location of the rings with a transparent window in cross section of the generated VB. Since the higher transmission in *y* direction appears only in a single ring (for $p=0$, $l=0$) and two rings (for $p=0$, $l\ne 0$), the birefringence is dominant in generation of the NMOR in these rings. Now, we are interested in determination of the two other higher NMOR rings, displayed in Fig. 2, which do not appear in the transmission profile. The lost high NMOR rings are related to the location of the absorption peaks shown in Fig. 3. As mentioned above, the maximum absorption is equal for both circular components, while their corresponding dispersion is largely different. Despite the large absorptions, the high NMOR occurred in those rings are due to mere birefringence which can be seen in Fig. 2. In the third row of Fig. 4, the evolution of the Rabi frequency of the LG control field is depicted for $y=0$. A line is seen in the diagrams that corresponds to $\Omega _c = \Delta _B$, which is crucial. Applying a static magnetic field causes the detuning of the transitions corresponding to the right- and left-circular components of the probe field to be shifted by $+\Delta _B$ and $-\Delta _B$, respectively. In addition, the AC stark effect due to the LG control field applies an additional detuning value, equal to $-\Omega _c$, to the $\Delta _{\pm }$^55^. Finally, we have $\Delta _+\rightarrow \Delta _++\Delta _B-\Omega _c$ and $\Delta _-\rightarrow \Delta _--\Delta _B-\Omega _c$. Since the circular components of the probe field in the absence of the static magnetic and control fields are assumed in resonance, the one-photon transition for the right- and left circular components, after applying fields, occurs only at $\Omega _c=\Delta _B$ and $\Omega _c=-\Delta _B$, respectively. It is well-known that the optical properties of a non-closed interaction loop system do not depend on the sign of the control field, so the absorption is the same for $\Omega _c=\pm \Delta _B$^56^. Fortunately, our analytical and numerical results show that the maximum absorption of both circular components occurs around $\Omega _c=\Delta _B$. In this regard, the exact locations of the maximum NMOR in the equal absorption peaks can be specified by the intensity profile of the LG control field. As a result, the condition $\Omega _c = \Delta _B$ determines the position of the perfect NMOR rings induced by mainly birefringence in absorption peaks of circular components, as shown in the third row of Fig. 3. So the contribution of birefringence in all rings with perfect NMOR, appeared in Fig. 2, is dominant and the dichroism has a negligible contribution in the generation of the VB. It should be noted that since each intensity for $p=0$ and $l\ne 0$ is observed in two radii of the LG field’s intensity profile, one can see that the inner and outer rings in the second row of Fig. 2 are generated in the rings with maximum absorption of circular components, while two others happen in transparency windows. The inner (outer) ring with the maximum NMOR for $p=0$ and $l=0$, corresponding to the higher (lower) control field intensity, is established in the transparent window (absorption peak).

Now, we use Stokes parameters to have another insight into understanding the state of polarization in the cross section of the generated VBs. Stokes parameters based on the electric field amplitude are a useful tool to describe and measure the optical polarization^57,58^. The normalized Stokes parameters are given with the four elements by^59^

10 $$\begin{aligned} S=(1 \quad S_1 \quad S_2 \quad S_3)^{Trans}. \end{aligned}$$

**Figure 5:**
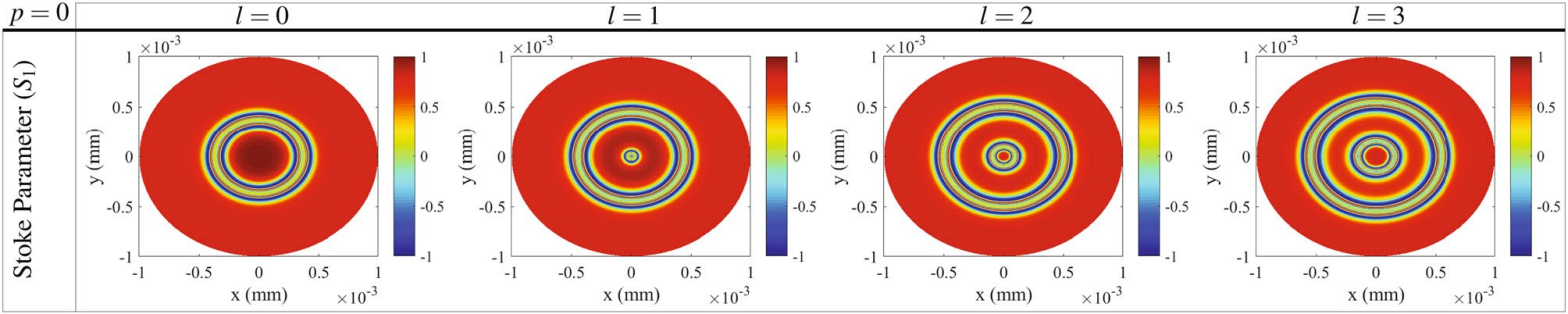
Profile of $S_1$ of Stokes parameters as a function of *x* and *y* for Gaussian and different modes of LG modes. The used parameters are those used in Fig. 2.

$S_1=(T_x - T_y)/S_0$, $S_2=(2\sqrt{T_x}\sqrt{T_y} cos\delta )/S_0$ and $S_3=(2\sqrt{T_x}\sqrt{T_y} sin\delta )/S_0$, where $S_0=T_x + T_y$ and $\delta =0$ for linear polarization. The superscript *Trans* stands for the transpose of the matrix. In our work, $T_{y}$ and $T_x$ are the intensities of the transmission in *y* and *x* directions, respectively. Since the polarization of linearly polarized transmitted field makes an angle $\phi$ with *x* direction, $S_1$ and $S_2$ lead to $S_1=\cos 2\phi$ and $S_2=\sin 2\phi$, respectively. So, it is expected that the parameter *S*1 represents the magnitude of NMOR angle in VB’s cross section. In Fig. 5, the profile of *S*1 is presented as a function of *x* and *y* for Gaussian and different modes of LG modes. The positions with values of − 1 and 1 show the positions of the perfect NMOR and the initial polarization, respectively, which are in good agreement with the results of the second row of Fig. 2.

Here, we are going to present the analytical expressions to understand the physics of the phenomena and the role of the different parameters in the evolution of the system. The analytical solutions for the transition coherences $\rho _{31}$ and $\rho _{32}$ in the weak probe field approximation are given by

11 $$\begin{aligned} \rho _{31}= & {} \frac{2Z\Delta ^{4}_{B}}{D}\Omega _{p+}+\frac{2\Delta ^{2}_{B}(iA+Z)\Omega ^{2}_{c}}{D}\Omega _{p+}+\frac{2iA\Omega ^{4}_{c}}{D}\Omega _{p+} \end{aligned}$$

12 $$\begin{aligned} \rho _{32}= & {} \frac{2ZA^2\Delta ^{2}_{B}}{D}\Omega _{p-}+\frac{i\Delta ^{2}_{B}(4\gamma +\Delta _B)\Omega ^{2}_{c}}{D}\Omega _{p-}+\frac{A^{*}(1+2i)\Omega ^{4}_{c}}{D}\Omega _{p-}, \end{aligned}$$

where $D=(A-i\Omega _{c})(-iA+\Omega _{c})(4ZA^{*}\Delta ^{2}_{B}+((8-2i)\gamma \Delta _B-\Delta ^{2}_{B})\Omega ^{2}_{c}+(1+4i)\Omega ^{4}_{c})$, $A=\gamma +i\Delta _{B}$ and $Z=\gamma +iA^{*}$. The coherence terms corresponding to the transitions $|1\rangle \leftrightarrow |2\rangle$, $|1\rangle \leftrightarrow |4\rangle$ and $|2\rangle \leftrightarrow |4\rangle$ do not play major roles and have been dropped in calculation of Eqs. (11, 12). The first terms are the direct responses of the medium to the circular components of the probe field. The second terms stand for the cross-Kerr effect through the three-photon transitions $|1\rangle \xrightarrow {\Omega _{p+}}|3\rangle \xrightarrow {\Omega _{c}}|4\rangle \xrightarrow {\Omega ^{*}_{c}}|3\rangle$ and $|2\rangle \xrightarrow {\Omega _{p-}}|3\rangle \xrightarrow {\Omega ^{*}_{c}}|4\rangle \xrightarrow {\Omega _{c}}|3\rangle$ for the right- and left-circular components of the probe field, respectively. The third terms correspond also to the cross-Kerr effect, but through the five-photon transitions for the right- $|1\rangle \xrightarrow {\Omega _{p+}}|3\rangle \xrightarrow {\Omega _{c}}|4\rangle \xrightarrow {\Omega ^{*}_{c}}|3\rangle \xrightarrow {\Omega _{c}}|4\rangle \xrightarrow {\Omega ^{*}_{c}}|3\rangle$ and for the left- $|2\rangle \xrightarrow {\Omega _{p-}}|3\rangle \xrightarrow {\Omega _{c}}|4\rangle \xrightarrow {\Omega ^{*}_{c}}|3\rangle \xrightarrow {\Omega _{c}}|4\rangle \xrightarrow {\Omega ^{*}_{c}}|3\rangle$ circular components of the probe field. Equations (11) and (12) are in good agreement with the numerical results and demonstrate the contribution of the different nonlinear effects on the obtained NMOR.

**Figure 6:**
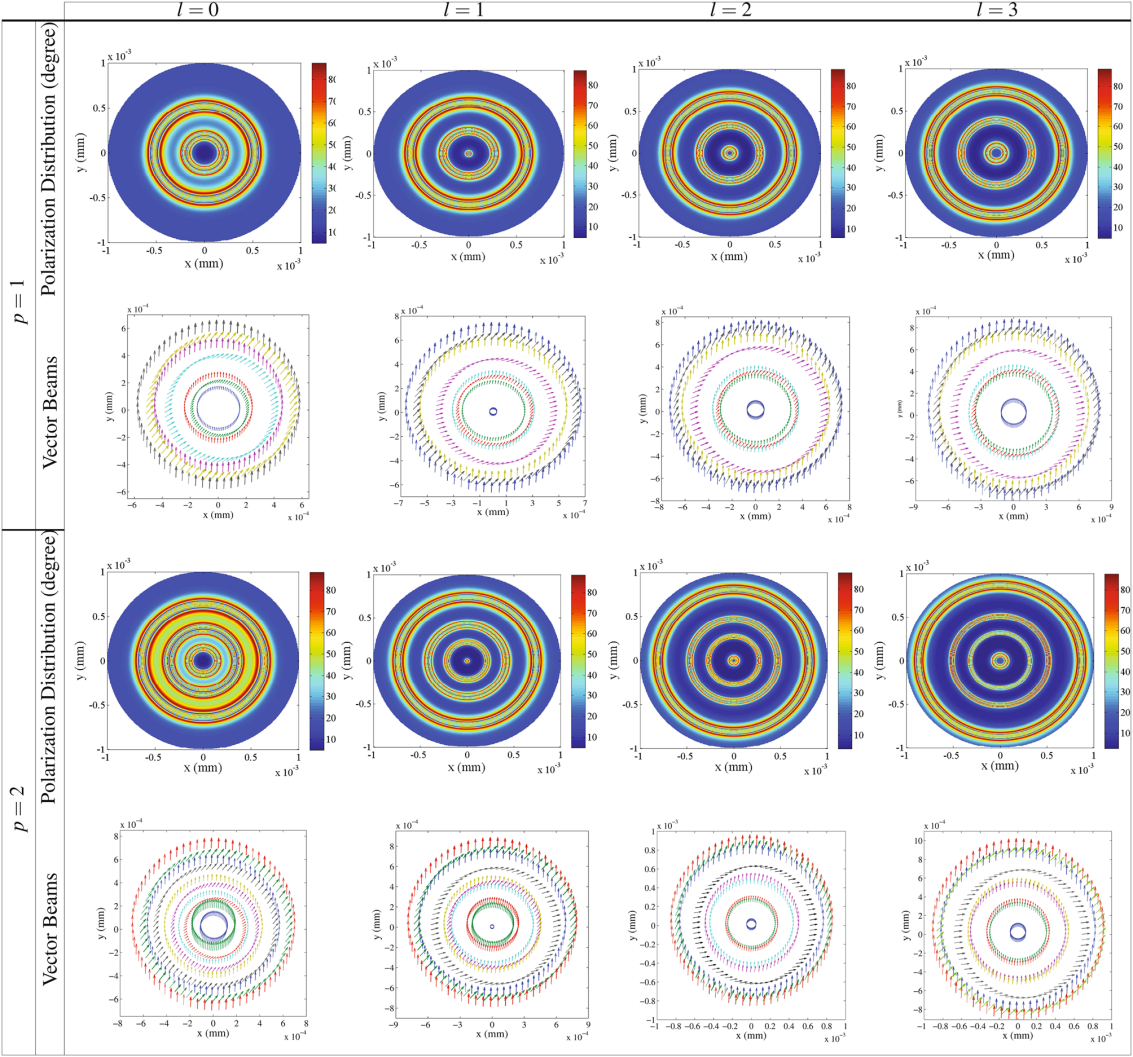
Polarization distribution of the transmitted probe field (first and third rows) in unit of degree and a schematic of the generated VB (second and fourth rows) as a function of *x* and *y* for Gaussian field $l=0$ and different modes of LG control field $l= 1$, $l= 2$ and $l= 3$ for the radial indices $p=1$ and $p=2$ of the LG field. The other parameters are those used in Fig. 2.

Now, we are going to investigate the effect of the radial index *p* on the generation of VBs. Overall, the effect of *p* has been rarely discussed on the optical phenomena^60^. However, it has been proved that consideration of *p* increases the information degree of freedom, allowing multi-dimensional quantum computing and encryption^61–63^. Figure 6 displays the effect of *p* on the spatial distribution of the polarization direction of VBs in unit of degree for $p= 1$ (first and second rows) and $p= 2$ (third and fourth rows) for different values of the OAM, i.e. $l=0$ (first column), $l=1$ (second column), $l=2$ (third column) and $l=3$ (fourth column) as a function of *x* and *y*. The other parameters are those used in Fig. 2. It is seen that for $p>0$ and $l=0$, the central part of the profiles has the least NMOR similar to the results of the Gaussian control field, but with a smaller radius than the case of $p=0$. However, the rest of the space possesses different polarizations so that they spatially vary by increasing the value of *p*. It is demonstrated that for $p>0$ and higher modes of the LG control field, the new regions which are limited between rings with higher NMOR angle are created with various polarizations distribution. Also, the number of these new regions increases by increasing the value of *p*. This implies that the generated VB contains a polarization distribution with more spatial variation than the case of $p=0$. Moreover, it is seen that by increasing the magnitude of *p*, the number of the rings with higher NMOR angle increases, leading to the generation of the VB with more nearly complete NMOR rings. It is worth to note that for any value of *p*, polarization distribution of the transmitted VBs still varies by increasing the OAM and extends to larger radii. A schematic of the wide polarization direction range of the generated VBs are presented in Fig. 6 for $p= 1$ (second row) and $p= 2$ (fourth row). The effect of *p* on the generation and polarization distribution of VBs can be seen in these schematic figures. Considering the direction of the initial polarization in *x* direction, one can see the amount of the NMOR angle in corss section of the transmitted VBs. It is resulted that *p* has a major role in generation and spatial distribution of VBs with a wide variety of new polarization directions induced by the NMOR technique. Thus, the radial index *p* provides extra capacity in space for optical communications.

**Figure 7:**
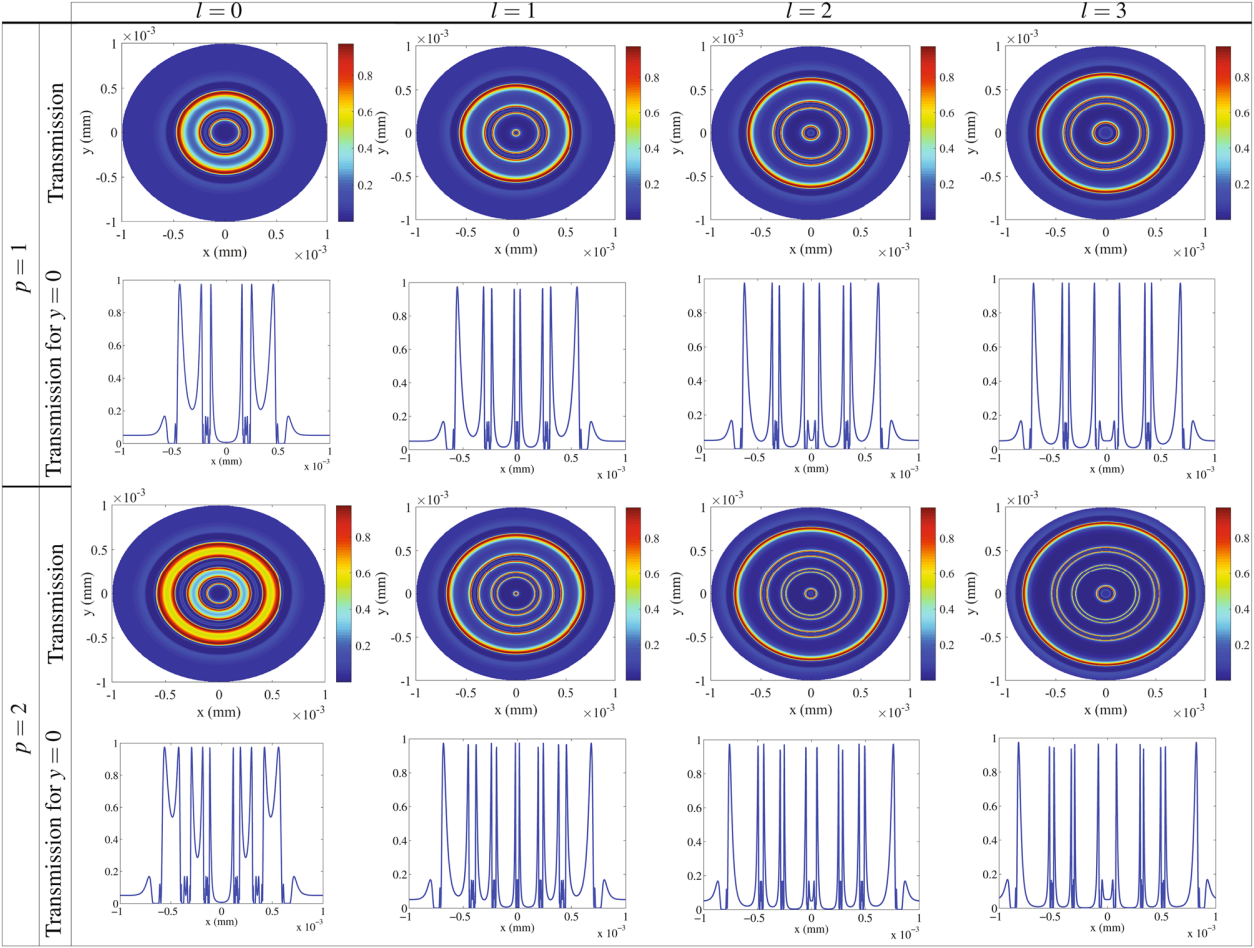
The *y*-polarized intensity distribution of the VB as a function of *x* and *y* for different OAMs of the radial indices $p=1$(first row) and $p= 2$ ( third row) of the LG control field. The corresponding perspective of the transmission of the generated VBs in $y=0$ for $p= 1$ (second row) and $p= 2$ (fourth row).

**Figure 8:**
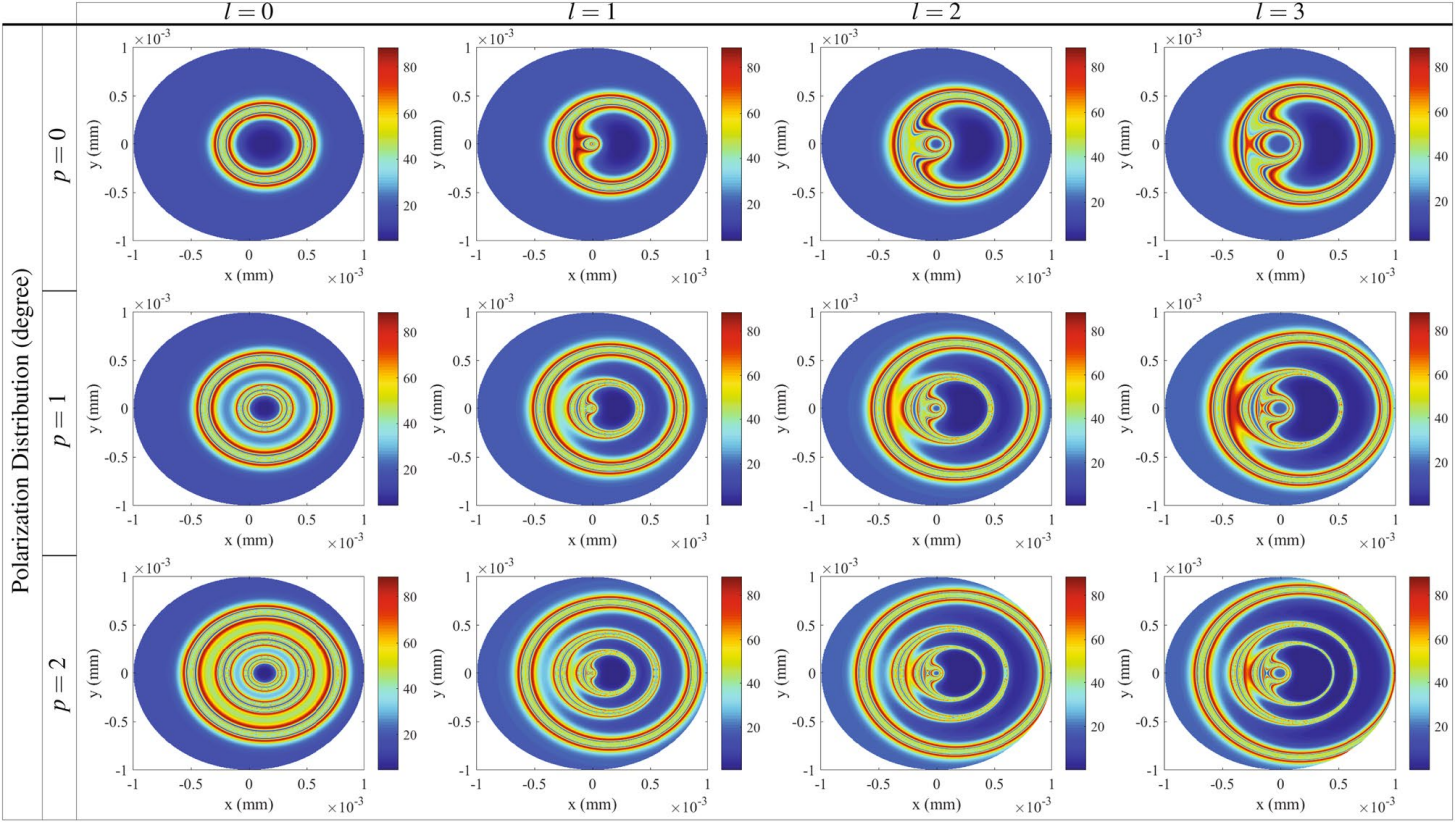
Polarization distribution of the VBs in unit of degree as a function of *x* and *y* for different OAMs of the radial indices $p=1$(first row), $p= 2$ (second row) and $p= 3$ (third row) of the aLG control field. the shift parameter a is equal to $a= 0.5 wLG$. Other parameters are those taken in Fig. 2 .

Here, we are going to show the intensity of the generated VB affected by *p*, which is a significant feature of VBs. Intensity of *y*-polarized transmission of VBs related to the radial indices $p= 1$ (first row) and $p= 2$ (third row) for different modes of the LG control field is presented in Fig. 7. It is shown that the generated VB has a certain intensity in different points of the space, and this intensity covers a wide range from the lowest to the highest intensity. Figure 7 shows that the intensity distribution of VBs can be also controlled by *p*. To clarify the obtained results, we display a corresponding perspective of the transmission of the generated VBs in $y=0$ for $p= 1$ (second row) and $p= 2$ (fourth row). The transmission peaks stand for the higher NMOR rings induced by the birefringence. So, other higher NMOR rings depicted in Fig. 6 are generated by the dichroism induced in the system. An investigation on Fig. 7 shows that *p* plays an important role in increasing the intensity of VBs. It is worth noting that the VBs with high intensity rings are generated in larger radii as *p* increases. Thus, the radial index *p* has a significant effect on the intensity distribution of the transmitted VBs. The generated VB in our results have an azimuthally symmetric of the polarization distribution which makes the work fall short on impact. Here, we are interested in breaking the azimuthally symmetric using non-coaxial LG beams which is made by imposing a transverse shift in the vortex center of symmetrical LG modes^64^. Assuming a shift by *a* along the positive *x* direction, Eq. (1) is transformed to the aLG beams amplitude as^65^

13 $$\begin{aligned} E_{c}(r,\varphi )=E_{0_{c}}\frac{w_{G}}{\sqrt{|l|!}w_{LG}} \left (\frac{\sqrt{2}r}{w_{LG}} \right)^{|l|}\times L^{|l|}_{p}(x)e^{-(r^2+a^2-2arcos\varphi )/w_{LG}^{2}}e^{il\varphi }, \end{aligned}$$

where $x=2(r^2+a^2-2arcos\varphi )/w_{LG}^{2}$. The polarization distribution of the generated VBs in unit of degree is shown in Fig. 8 for $p=0$ (first row), $p=1$ (second row) and $p=2$ (third row) for different OAMs of the aLG control field as a function of x and y. Through the results, $a= 0.5 w_{LG}$ and the other parameters are those taken in Fig. 2. It is seen that the azimuthal symmetry of the polarization distribution of the VBs is broken using aLG. For $l=0$, the polarization distribution is just shifted without any asymmetry. By applying different modes of the aLG control field, the polarization is no longer azimuthally symmetric in the cross section of the VBs. By increasing the value of the OAM, it is shown that the polarization distribution becomes more asymmetric. Moreover, the radial index of the aLG control fields is another useful parameter in increasing the asymmetry of the polarization distribution. It should be noted that since the generated VB is due to the effect of the intensity profile of the LG beam and the helical phase front structure via interference does not have any contribution to the generated VB, it cannot be destroyed during propagation through the medium. Let us calculate the Fresnel’s diffraction integral to investigate the stability of the generated VBs during its propagation in free space after leaving the medium. Our calculations confirm that the propagation of the generated VBs does not affect the polarization distribution, leading to the stable VBs.

## Conclusion

In summary, the LG beam was presented to generate and control VB via NMOR. Our scheme was aimed to simplify the generation and control of VBs with respect to the previous methods. The basis of our work was the NMOR of the polarization direction of a linearly polarized probe field passing through an inverted Y-type four-level quantum system subjected to a LG control field and a static magnetic field. It was shown that the polarization of the transmitted field varies spatially by the OAM of the LG control field, leading to an azimuthally symmetric polarization distribution and generation of VB. In addition, we found that the intensity of VBs can be easily controlled by the characteristics of the LG control field. We demonstrated that the radial index *p* has a major role in more spatially distribution of VB with different polarization directions; so that by increasing *p*, the VB with more number of higher NMOR rings were generated with higher intensity of transmission. The contribution of the direct response of the medium, as well as the different nonlinear cross-Kerr effect, in generation of the VBs was determined by the analytical results. Besides, we showed that the polarization distribution of VB is due to mainly birefringence induced in the system. Finally, aLG field was used for breaking the symmetry of the polarization distribution of the VBs to increase their efficiency. It was shown that by applying the aLG control field, azimuthally asymmetric polarization distribution is achieved and the degree of asymmetry can be controlled by the OAM and radial index of the aLG control field. The simple generation method, controllably spatial distribution and intensity profile of VBs in our work provide an excessive capacity in optical communication and optical networking.
